# Invasive Fungal Infections in the Neonatal Intensive Care Unit

**DOI:** 10.7759/cureus.79620

**Published:** 2025-02-25

**Authors:** Sabina Terzic, Alma Zgalj

**Affiliations:** 1 Pediatrics, Clinical Center University of Sarajevo, Sarajevo, BIH; 2 General Medicine, Public Institution Health Centre of Sarajevo Canton, Sarajevo, BIH

**Keywords:** invasive fungal infection, morbidity, mortality, neonate, nicu

## Abstract

Background

Due to the great burden of morbidity and mortality, invasive fungal infections (IFIs) are a major concern in neonatal intensive care units (NICUs) worldwide. This study aimed to evaluate the epidemiological and clinical characteristics, type of pathogen, risk factors, laboratory parameters, treatment, and clinical outcome associated with IFIs in a NICU within a three-year period.

Methodology

This study was conducted at the NICU, Pediatric Clinic, Clinical Centre of the University of Sarajevo from January 2020 to December 2022. A total of 22 infants were diagnosed with IFIs. Clinical data of the infants were collected from medical histories, including gestational age, gender, birth weight, perinatal conditions, major diseases, type and site of fungal isolate, time of onset of infection, clinical and laboratory findings, antifungal prophylaxis, antifungal therapy, broad-spectrum antibiotic therapy, clinical interventions, associated bacterial sepsis, total duration of hospitalization, and clinical outcomes. The study was approved by the Ethics Committee of Sarajevo University, Faculty of Medicine, Clinical Center University of Sarajevo. All cultures were monitored using an automated culture system. Passages to blood agar and Sabouraud dextrose agar were performed. Absolute numbers and percentages were used to describe categorical data, and median and interquartile ranges were used to describe numerical data due to data distribution that deviated from normal. The chi-square test or Fisher’s exact test was used to analyze differences in categorical variables. The Mann-Whitney U-test and the Wilcoxon signed-rank test were used to analyze the differences in numerical variables between the observed groups. P-values <0.05 were considered statistically significant. Statistical analyses were performed using SPSS version 25.0 (IBM Corp., Armonk, NY, USA).

Results

Between January 2020 and December 2022, a total of 22 infants had IFIs in the NICU, with an overall incidence of 11.5 per 1,000 admissions per year. *Candida albicans* was the most frequently isolated species (54.5%). The most important risk factors for IFIs in the NICU were long-term use of broad-spectrum antibiotics, parenteral nutrition, central venous catheter (CVC), mechanical ventilation (MV), birth weight <1,500 g, and prematurity. A significant increase in the median C-reactive protein and a decrease in the median platelet count was recorded between admission and the time of pathogen isolation. The overall mortality rate was 27.3% with significantly higher mortality in patients with *C. albicans* compared to other *Candida* species.

Conclusions

In this study, *C. albicans* was the most frequent causative agent of IFI (54.5%). The most important risk factors for IFIs in the NICU were long-term use of broad-spectrum antibiotics, parenteral nutrition, CVC, MV, birth weight <1,500 g, and prematurity. The mortality rate was significantly higher in patients infected with *C. albicans* than with non-albicans species.

## Introduction

Invasive fungal infections (IFIs), where fungi are isolated from blood and other sterile sites and tissues showing invasion into specific organs, present a major concern in neonatal intensive care units (NICUs) [1]. They are the third most common cause of late-onset sepsis, following coagulase-negative staphylococci and Gram-negative bacilli [2,3]. The incidence of invasive candidiasis in NICUs is increasing due to the increased survival and intensive care of extremely immature infants. It ranges from 2.6% to 13.2% in very low birth weight (VLBW) infants (1,500-1,000 g) and from 6.6% to 26.0% in extremely low birth weight (ELBW) infants (<1,000 g) [4]. Neonatal invasive candidiasis is associated with high mortality, with an overall estimated rate of approximately 20%, with up to 50% in ELBW infants [5]. Benjamin et al. reported an overall mortality of 34% for ELBW infants with invasive candidiasis compared with 14% for ELBW infants without invasive candidiasis [6]. Due to prolonged intensive care, premature infants are exposed to various risk factors for IFIs, such as prolonged use of broad-spectrum antibiotics, immature immune system, central venous lines, mechanical ventilation (MV), abdomen surgery, total parenteral nutrition, and inhibitors of gastric acidity [7-10]. *Candida albicans* is the most common pathogen associated with neonatal fungal infections, and *Candida parapsilosis* is the second most common yeast species isolated from bloodstream infections [11,12]. This study aimed to evaluate the epidemiological and clinical characteristics of IFIs in newborns and determine the type of pathogens, risk factors, laboratory parameters, and the therapeutic approach to infection.

## Materials and methods

This study was conducted at the NICU in the Pediatric Clinic, Clinical Centre of the University of Sarajevo from January 2020 to December 2022. A total of 22 of 1,922 neonates were diagnosed with IFIs according to clinical findings and positive cultures (blood, cerebrospinal fluid (CSF), and/or urine). All neonates with diagnosed IFIs were included in the study. The exclusion criteria were the lack of confirmed IFIs. Clinical data of the neonates were collected from medical histories, including gestational age, gender, birth weight, perinatal conditions (delivery mode, premature rupture of membranes, antenatal corticosteroids), major diseases, type of fungal isolate and the place of isolation, time of onset of infection, clinical findings, laboratory findings, antifungal prophylaxis, antifungal therapy, broad-spectrum antibiotic therapy, clinical interventions (MV, central venous lines, total parenteral nutrition) and operation sites, associated bacterial sepsis, total duration of hospitalization, and clinical outcomes. The study was approved by the Ethics Committee of Sarajevo University, Faculty of Medicine, Clinical Center University of Sarajevo (approval number: 50-30-5-56/06/22).

Microbiology

Blood cultures were obtained from peripheral veins by sterile technique, CSF was obtained by lumbar puncture, and urine by urethral catheterization. Urinary tract infection was defined as the growth of a single organism at ≥10^4^ CFU/mL in urine collected by catheterization. All cultures were monitored using an automated culture system. Passages to blood agar and Sabouraud dextrose agar were performed.

Statistical analysis

The data were initially collected and entered into a Microsoft Excel 2017 (Microsoft Corp., Redmond, WA, USA) master sheet. Absolute numbers and percentages were used to describe categorical data, while median and interquartile range (IQR) were used to describe numerical data due to data distribution that deviated from normal. The chi-square test or Fisher’s exact test was used to analyze differences in categorical variables. The Mann-Whitney U-test and the Wilcoxon signed-rank test were used to analyze the differences in numerical variables between the observed groups. P-values <0.05 were considered statistically significant. Statistical analyses were performed using SPSS version 25.0 (IBM Corp., Armonk, NY, USA).

## Results

A total of 1,922 neonates were admitted to the NICU between January 2020 and December 2022. During the study period, we identified 22 (1.14%) neonates with IFIs, of whom 12 (54.5%) were female. The overall incidence of IFIs over the three-year period was 11.5 per 1,000 NICU admissions annually, with the highest incidence rate in 2021. (18.9 per 1,000 admissions) and the lowest incidence rate in 2020 (5.0 per 1,000 admissions). The median gestational age at birth was 30.5 weeks (range = 28-40 weeks). Early preterm infants (25-34 weeks of gestation) were more common compared to term infants (37-40 weeks of gestation) and late preterm infants (34+0 to 36+6 weeks of gestation) at 54.5%, 31.8%, and 13.6%, respectively. The median birth weight was 1,372.5 g (range = 965-2,472.5 g). Most infants were ELBW (31.8%).

*Candida* spp. was the cause of IFIs in all 22 subjects. *C. albicans* was the most frequently isolated species (54.5%), followed by *Candida glabrata* (27.3%), *C. parapsilosis* (27.3%), *Candida lusitaniae* (4.5%), and unidentified *Candida* spp. (4.5%). In four cases, two types of *Candida* were identified simultaneously on the same fungal culture (in two patients, *C. glabrata *with *C. parapsilosis*, and in the remaining, *C. albicans* with *C. parapsilosis* and *C. parapsilosis* with *C. lusitaniae*).

The most common site of isolation of the pathogen was blood (86.4%), followed by urine (9.1%), and abdominal drain (4.5%). The type of *Candida* that was present in urine and blood was present in other samples (stool and urine) in 9.1% of patients. The median age at onset of IFIs was 20 days (range = 11-30.5 days). There was no statistically significant difference in the mean age at onset of IFIs between patients with *C. albicans* and non-albicans *Candida* (p = 0.824). The rates of occurrence of the associated risk factors are presented in Table 1.

**Table 1:** Risk factors of associated with neonatal invasive fungal infections (IFIs).

Risk factor	Number of IFI cases, n (%)
Birth weight <1,500 g	12 (54.5)
Gestation age ≤28 weeks	7 (31.8)
Central venous catheter	21 (95.5)
Mechanical ventilation	12 (54.5)
No antifungal prophylaxis	21 (95.5)
Antibiotic use	22 (100)
Underlying disease	21 (95.5)
Antenatal corticosteroid therapy	7 (31.8)
Premature rupture of membranes	5 (22.7)
Vaginal delivery	6 (27.3)

Birth weight <1,500 g was present in 54.5% of neonates, and gestational age ≤28 weeks in 31.8% of neonates. Central venous catheter (CVC) was used in almost all patients (95.5%), and MV was used in 54.5% of patients. Almost all of our cases were on parenteral nutrition (21/22), with partial enteral nutrition (22/22). Only 4.5% of neonates received antifungal prophylaxis before the development of IFIs. All infants received two or more broad-spectrum antibiotics by the time of IFI onset. The most commonly used antibiotics were ampicillin (86.4%) and aminoglycosides (amikacin and gentamicin) (86.4%), vancomycin (81.8%) and meropenem (81.8%), followed by metronidazole (18.2%), polypeptides (colistin) (13.6%), and first-generation cephalosporins (cefazolin) (4.5%). The median total duration of antibiotic therapy by the time of IFI onset was 14 days (range = 11-17 days).

The most common underlying disease in patients with IFI was respiratory distress syndrome (54.5%). Associated bacterial sepsis was recorded in 12 (54.5%) cases, and Gram-negative rods were the most common bacterial causative agents (n = 10), and the rest were gram-positive cocci (n = 2). The most frequently recorded neurological diseases and conditions in our patients were intraventricular hemorrhage, thalamostriate vasculopathy, hydrocephalus, and periventricular leukomalacia. A surgical procedure preceding the IFI was performed in 10 (45.5%) patients, of which abdominal surgery was the most common (27.3%). These surgical conditions included ileus, necrotizing enterocolitis with perforation, gastroschisis, and volvulus.

On analyzing potential risk factors, we did not find statistically significant differences between patients infected with *C. albicans* and non-albicans *Candida*, except in the case of MV, where patients with *C. albicans* were significantly more likely to receive MV (Table 2).

**Table 2:** Risk factors for Candida albicans vs. non-albicans Candida spp.

Risk factors			Candida albicans	Non-albicans *Candida* spp.	P-value
Mechanical ventilation	No	n	3	7	χ^2^ = 5.051, p = 0.035
		%	30.0	70.0	
	Yes	n	8	2	
		%	80.0	20.0	
Surgery	Yes	n	5	4	χ^2^ = 0.002, p = 0.658
		%	55.6	44.4	
	No	n	6	5	
		%	54.5	45.5	
Gestation age	Premature (<37 weeks)	n	8	6	χ^2^ = 0.087, p = 0.574
		%	57.1	42.9	
	Term newborn (≥37 weeks)	n	3	3	
		%	50.0	50.0	
Birth weight	<1,500 g	n	6	6	χ^2^ = 0.303, p = 0.465
		%	50.0	50.0	
	>1,500 g	n	5	3	
		%	62.5	37.5	
Associated bacterial sepsis	No	n	6	3	χ^2^ = 0.900, p = 0.406
		%	66.7	33.3	
	Yes	n	5	6	
		%	45.5	54.5	

In this analysis, the case with unidentified *Candida* spp. and the case with mixed *C. albicans* and non-albicans *Candida* infection were excluded.

At the time of sampling the culture in which IFI was proven, almost all respondents, 95.5% (n = 21), were symptomatic. Clinical findings were respiratory symptoms such as dyspnea, apnea, desaturation, fever, worsening in general condition, pallor and marmorization, lethargy, and abdominal distension. In two cases, multiple abscesses were present on ultrasound of the central nervous system. The median total duration of hospitalization was 61.5 days (range = 35.75-86 days). We measured C-reactive protein (CRP), the number of leukocytes (white blood cells), and platelets on admission to the NICU and compared them with those at the time of isolation of the causative agent. There was a significant increase in the median CRP (4.6 vs. 71.6, p = 0.001), and a significant decrease in the median platelet count (234.5 vs. 119.0, p = 0.032) between admission and isolation of the causative agent. There was no statistically significant difference in the median number of leukocytes on admission compared to the isolation of the causative agent (p = 0.614) (Table 3).

**Table 3:** Laboratory findings.

Laboratory findings	Median		Mean		P-value
	On admission	At isolation	On admission	At isolation	
C-reactive protein	4.6	71.6	24.4 ± 44.2	92.2 ± 68.4	0.001
White blood cells	10.3	13.0	13.2 ± 8.6	13.6 ± 5.9	0.614
Platelets	234.5	119.0	230.7 ± 86.9	157.4 ± 113.6	0.032

Fluconazole was the first-line antifungal therapy in all 22 patients. In three cases, the first-line antifungal therapy had to be changed. Voriconazole was the definitive therapy in two cases, and amphotericin B in one case. The median duration of antifungal therapy in survivors was 34.5 days (range = 24-40.5 days). Six patients died during antifungal therapy (27.3%). Among the deceased patients, four were infected with *C. albicans*, one with *C. albicans* and *C. parapsilosis*, and one with *C. lusitaniae* and *C. parapsilosis*. All deceased patients were treated with fluconazole, and in one case, fluconazole was replaced with voriconazole. Five patients died within 30 days of the onset of IFI. IFI directly contributed to the death of these patients. Gender, maturity, birth weight, MV, associated bacterial sepsis, and surgery were not associated with better or worse clinical outcomes (p > 0.05). The outcome of infants who received a single drug treatment was 73.7% recovery compared with infants whose antifungal therapy was switched wherein 66.7% had recovered. The difference was not statistically significant (p = 0.636). In addition, 12 patients had persistent bacterial sepsis, of whom four died. The overall outcome of infants with IFI was 72.7% recovery and 27.3% death.

## Discussion

In recent years, IFIs have been an increasing problem in NICUs. The great progress of medicine in intensive care has contributed to the increased survival of extremely premature infants but also to an increased incidence of IFIs [4]. For these reasons, this study aimed to examine the epidemiological and clinical characteristics of IFIs in the NICU of the Pediatric Clinic of the University Hospital of Sarajevo.

The most common cause of IFI in 22 subjects in this study was *C. albicans* (54.5%). Several studies agree with our results that *C. albicans* is still the dominant cause of IFIs [9,13-15]. An increasing number of studies in the last few years have reported an increased frequency of non-albicans *Candida* spp., and in some, non-albicans *Candida* and/or *C. parapsilosis* have been shown to be the leading causes of IFIs in the NICU [16,17]. Although *C. albicans* was the dominant cause of IFIs in our research, we also observed a significant increase in non-albicans *Candida* infections (among them both *C. parapsilosis* and *C. glabrata*), which increased from zero cases in 2020 to five cases in 2021.

In this study, 86.4% of *Candida* spp. were isolated from blood. This is consistent with findings of similar studies that reported candidemia as the most common finding in IFIs [18,19]. The median age at onset of IFIs was 20 days, which is almost the same as that reported by Ishiwada et al. [14].

Observing the relationship between gender and the development of IFIs, a slight dominance of females was observed compared to males at 12 vs. 10. In some studies, there was a slight dominance of males [16,20]. The median gestational age and birth weight of newborns with IFIs in our study were 30.5 weeks and 1,372.5 g, respectively, which is comparable to most other studies [12,15,17].

The incidence of IFIs varies significantly between centers and over the years, and the increase in incidence has certainly been contributed by the increased survival rate of premature infants and advances in medical technology and therapy, which predispose the development of IFIs in this vulnerable group. In our study, the incidence varied over the years, with the highest at 18.9 per 1,000 admissions in 2021, and the lowest at 5.0 per 1,000 admissions in 2020. The overall incidence over a three-year period was 11.5 per 1,000 admissions per year, which was similar to the incidence reported by Almazer et al. in a five-year study in Saudi Arabia (9.9 per 1,000 admissions per year) [15]. A study conducted in Mexico showed a significantly lower incidence of 2.27 per 1,000 live births [7], in contrast to a study in Panama, which reported a higher incidence (20 per 1,000 admissions per year) than that observed in our study [16]. Possible explanations for these discrepancies include demographic differences, the development of clinical centers, and the irrational use of broad-spectrum antibiotics.

The main risk factors for IFIs are long-term use of broad-spectrum antibiotics including third-generation cephalosporins, CVC, MV, parenteral nutrition, immunological immaturity, and birth weight <1,500 g [14,21]. We investigated the frequency of these established risk factors for IFI.

Exposure to broad-spectrum antibiotics, especially carbapenems, third-generation cephalosporins, vancomycin, and beta-lactamase inhibitors, is one of the major risk factors for the development of IFIs among NICU patients [7,15,16,20]. In this study, all infants were exposed to broad-spectrum antibiotics by the time of IFI onset, and the most commonly used antibiotics were aminoglycosides (86.4%), ampicillin (86.4%), vancomycin (81.8%), and meropenem (81.8%).

CVC and parenteral nutrition are known risk factors for IFIs and were present in most patients in similar studies [14,15,20]. In this study, CVC and parenteral nutrition were present in 21/22 patients (95.5%).

Antifungal prophylaxis with fluconazole is a protective factor against IFIs in NICU patients. Several studies have shown that fluconazole prophylaxis reduced *C. albicans* and non-albicans *Candida* colonization, reduced mortality, and improved outcomes in ELBW infants [22-24]. In this study, only one patient was on fluconazole prophylaxis before the development of IFI. In a group of infants at high risk for IFIs, the potential benefit of prophylaxis is clear, and its use in this group outweighs the concerns of antifungal toxicity and possible resistance.

MV is another risk factor for the development of IFI, and the risk is proportionally related to its duration [7,15]. In our study, invasive MV was also present in more than half of the subjects (54.5%).

Prematurity is recognized as the most common underlying condition among newborns with candidemia, and the majority of newborns were VLBW or ELBW [21]. Caparó Ingram et al. reported 31.3% of newborns with gestational age <28 weeks and 67.9% of newborns with birth weight <1,500 g, which is similar to our results of 31.8% in patients ≤28 weeks and 54.5% of birth weight <1,500 g [16].

Other risk factors such as corticosteroid therapy for the mother (7/22), vaginal delivery (6/22), and premature rupture of the membranes (5/22) were recorded in <50% of cases with IFIs in our study, which was also reported in a study conducted in Japan [14].

Underlying diseases without ELBW were present in 95.5% of our newborns, of which respiratory diseases were the most prevalent with a prevalence of 54.5%, along with associated bacterial sepsis in 54.5%, followed by neurological diseases in 50%. In the study by Hsu et al., similar to this study, the most prevalent comorbid conditions were respiratory diseases at 53.1% [20]. Almazer et al reported neurological diseases as the most common underlying diseases with a prevalence of 48.3% [15].

Bacterial infections exhaust the patient’s immune response, making them susceptible to secondary fungal infections. In this study, bacterial sepsis was present in 12 (54.5%) patients, with Gram-negative rods as the most common causative agents (n = 10), mainly *Klebsiella*, and the rest with Gram-positive cocci (n = 2). This was even higher than what was reported by Chen et al. in Taiwan, where bacterial sepsis was observed in 10.1%, also with Gram-negative rods as the dominant causative agents, followed by Gram-positive cocci [12]. Another study found that neonates with IFIs had bacterial sepsis more frequently than controls (43.5% vs. 15.3%; p < 0.001), with *Klebsiella pneumoniae* being the most common causative agent [7].

Surgical interventions are another risk factor for the development of IFIs based on the disrupted skin and epithelial barrier and the consequent penetration of pathogens into the bloodstream. Surgery before diagnosis was the most common underlying disease in newborns, and abdominal surgery was the most common site of surgery (26%) [9]. In our study, surgical procedures were also among the leading underlying diseases (45.5%), with abdominal procedures being the most common (27.3%).

Longer NICU stays usually involve invasive procedures and their risk factors for IFI. The median total length of hospital stay in this study was 61.5 days, and a similar finding was reported in an American study, where the median total length of hospitalization of 63 days [9].

Analysis of potential risk factors found no statistically significant difference between patients infected with *C. albicans* and non-albicans *Candida*, except in the case of MV, which was significantly more often used in patients with *C. albicans*. The same results were reported by Lona-Reyes et al. [7].

Neonatal sepsis, especially IFI, is associated with certain laboratory findings. A study conducted in China showed that the platelet count is an economical and effective predictor of IFI in premature infants and that IFI should be suspected if the platelet count is <157 × 10^9^/L [25]. A statistically significant increase in the median CRP and a significant decrease in the median platelet count were reported between admission and isolation of the causative agent [15]. The same was recorded in our research.

Fluconazole remains the most commonly prescribed initial therapy for IFIs, followed by amphotericin B and caspofungin [20,26]. Fluconazole was the first-line drug of choice in all of our patients with IFIs, and in three cases, it had to be switched to voriconazole and amphotericin B. There was no statistically significant difference in outcomes between infants who received a single drug throughout treatment compared with infants in whom antifungal therapy had to be switched. The median duration of antifungal therapy in survivors was 34.5 days, which is almost the same as in a study conducted in Japan (34 days) [14].

We measured clinical outcomes as recovery and death. Gender, maturity, birth weight, MV, associated bacterial sepsis, and surgery were not associated with better or worse clinical outcomes (p > 0.05), which was also reported in a similar study [15].

In this study, mortality was significantly higher in *C. albicans* than in non-albicans *Candida* spp. However, Ahangarkani et al. found a higher mortality rate in non-albicans *Candida* spp. [27]. In a multicenter study conducted in Portugal, *C. albicans* was responsible for all deaths due to IFIs, and the reported zero mortality rate for *C. parapsilosis* is consistent with the evidence that *C. parapsilosis* has a lower virulence and is less invasive than *C. albicans* [11,28]. The fact that IFIs have a significant impact on mortality in the newborn period was also shown by a multicenter European study, which reported significantly higher mortality rates of candidemia among patients admitted to neonatal or pediatric intensive care units than to general and other pediatric and children’s surgical wards [29].

IFIs are caused by pre-existing underlying diseases, non-specific symptoms, and often poor general conditions. This explains why the disease is often suspected, diagnosed, and treated late in most cases, leading to a high mortality rate. It is often questioned whether mortality is directly related to IFIs or the disease for which the patient was primarily admitted to the NICU. The mortality rate attributable to candidemia was reported by Chen et al. to be 24.2% [12]. In Japan, Ishiwada et al. reported a mortality rate of 17.4% [14]. A much higher mortality rate than the previously mentioned ones was recorded in Iran at 52% [27]. In our study, the overall mortality rate was 27.3%.

There are several limitations of this study, such as the retrospective method of data collection, the small sample size due to low incidence, the focus on one health center, and the policy of our institution according to which we did not implement the prophylaxis of fungal infections.

## Conclusions

This study showed that the frequency of IFIs in the NICU at the Pediatric Clinic, Clinical Center University of Sarajevo in the examined period was 11.5 cases per 1,000 admissions per year. The overall mortality rate of IFIs was 27.3% and was significantly higher in patients infected with C. albicans than with non-albicans Candida species. The most important risk factors for IFIs in the NICU were long-term use of broad-spectrum antibiotics, parenteral nutrition, CVC, MV, birth weight <1,500 g, and prematurity. Statistically significant increases in CRP and a decrease in the number of platelets at the moment of isolation of the causative agent were recorded compared to the values before the development of IFIs. Future studies should focus on validating the Candida score and determining preventive interventions for IFIs in NICU patients.

## References

[1] Kwarteng Owusu S (2022). Invasive fungal infections. Afr J Thorac Crit Care Med.

[2] Hundalani S, Pammi M (2013). Invasive fungal infections in newborns and current management strategies. Expert Rev Anti Infect Ther.

[3] Stoll BJ, Hansen N, Fanaroff AA (2002). Late-onset sepsis in very low birth weight neonates: the experience of the NICHD Neonatal Research Network. Pediatrics.

[4] Aliaga S, Clark RH, Laughon M (2014). Changes in the incidence of candidiasis in neonatal intensive care units. Pediatrics.

[5] Pana ZD, Roilides E, Warris A, Groll AH, Zaoutis T (2017). Epidemiology of invasive fungal disease in children. J Pediatric Infect Dis Soc.

[6] Benjamin DK Jr, Stoll BJ, Gantz MG (2010). Neonatal candidiasis: epidemiology, risk factors, and clinical judgment. Pediatrics.

[7] Lona-Reyes JC, Gómez-Ruiz LM, Cordero-Zamora A, Cortés-González SI, Quiles-Corona M, Pérez-Ramírez RO, Pinto-Macedo H (2022). Incidence and factors associated with invasive candidiasis in a neonatal intensive care unit in Mexico. An Pediatr (Engl Ed).

[8] Dubbink-Verheij GH, Bekker V, Pelsma IC (2017). Bloodstream infection incidence of different central venous catheters in neonates: a descriptive cohort study. Front Pediatr.

[9] Benedict K, Roy M, Kabbani S (2018). Neonatal and pediatric candidemia: results from population-based active laboratory surveillance in four US locations, 2009-2015. J Pediatric Infect Dis Soc.

[10] Manzoni P, García Sánchez R, Meyer M (2018). Exposure to gastric acid inhibitors increases the risk of infection in preterm very low birth weight infants but concomitant administration of lactoferrin counteracts this effect. J Pediatr.

[11] Baptista MI, Nona J, Ferreira M (2016). Invasive fungal infection in neonatal intensive care units: a multicenter survey. J Chemother.

[12] Chen YN, Hsu JF, Chu SM (2022). Clinical and microbiological characteristics of neonates with candidemia and impacts of therapeutic strategies on the outcomes. J Fungi (Basel).

[13] Ting JY, Roberts A, Synnes A, Canning R, Bodani J, Monterossa L, Shah PS (2018). Invasive fungal infections in neonates in Canada: epidemiology and outcomes. Pediatr Infect Dis J.

[14] Ishiwada N, Kitajima H, Morioka I, Takeuchi N, Endo M, Watanabe A, Kamei K (2018). Nationwide survey of neonatal invasive fungal infection in Japan. Med Mycol.

[15] Almazer Y, Ashaq L, Aljohani E (2019). Invasive candidiasis in neonatal intensive care unit. J Pediatr Neonat Disord.

[16] Caparó Ingram E, Vásquez Vega M, Norero X, Sáez-Llorens X, DeAntonio R, Rodríguez Barría E (2019). [Risk factors and lethality associated with neonatal candidemia in a neonatal unit]. Rev Chil Pediatr.

[17] Caggiano G, Lovero G, De Giglio O, Barbuti G, Montagna O, Laforgia N, Montagna MT (2017). Candidemia in the neonatal intensive care unit: a retrospective, observational survey and analysis of literature data. Biomed Res Int.

[18] Agarwal RR, Agarwal RL, Chen X, Lua JL, Ang JY (2017). Epidemiology of invasive fungal infections at two tertiary care neonatal intensive care units over a 12-year period (2000-2011). Glob Pediatr Health.

[19] Ezenwa BN, Oladele RO, Akintan PE, Fajolu IB, Oshun PO, Oduyebo OO, Ezeaka VC (2017). Invasive candidiasis in a neonatal intensive care unit in Lagos, Nigeria. Niger Postgrad Med J.

[20] Hsu JF, Lai MY, Lee CW (2018). Comparison of the incidence, clinical features and outcomes of invasive candidiasis in children and neonates. BMC Infect Dis.

[21] De Rose DU, Santisi A, Ronchetti MP (2021). Invasive Candida infections in neonates after major surgery: current evidence and new directions. Pathogens.

[22] Hanna Pharm M, Mazkereth R (2021). Which patient in the neonatal intensive care unit should receive antifungal prophylaxis therapy?. Isr Med Assoc J.

[23] Robati Anaraki M, Nouri-Vaskeh M, Abdoli Oskoei S (2021). Fluconazole prophylaxis against invasive candidiasis in very low and extremely low birth weight preterm neonates: a systematic review and meta-analysis. Clin Exp Pediatr.

[24] Autmizguine J, Smith PB, Prather K (2018). Effect of fluconazole prophylaxis on Candida fluconazole susceptibility in premature infants. J Antimicrob Chemother.

[25] Yang YC, Mao J (2018). Value of platelet count in the early diagnosis of nosocomial invasive fungal infections in premature infants. Platelets.

[26] Ferreras-Antolín L, Sharland M, Warris A (2019). Management of invasive fungal disease in neonates and children. Pediatr Infect Dis J.

[27] Ahangarkani F, Shokohi T, Rezai MS (2020). Epidemiological features of nosocomial candidaemia in neonates, infants and children: a multicentre study in Iran. Mycoses.

[28] Gonia S, Archambault L, Shevik M (2017). Candida parapsilosis protects premature intestinal epithelial cells from invasion and damage by Candida albicans. Front Pediatr.

[29] Warris A, Pana ZD, Oletto A (2020). Etiology and outcome of candidemia in neonates and children in Europe: an 11-year multinational retrospective study. Pediatr Infect Dis J.

